# Green tea extracts containing epigallocatechin-3-gallate modulate facial development in Down syndrome

**DOI:** 10.1038/s41598-021-83757-1

**Published:** 2021-02-25

**Authors:** John M. Starbuck, Sergi Llambrich, Rubèn Gonzàlez, Julia Albaigès, Anna Sarlé, Jens Wouters, Alejandro González, Xavier Sevillano, James Sharpe, Rafael De La Torre, Mara Dierssen, Greetje Vande Velde, Neus Martínez-Abadías

**Affiliations:** 1 Department of Anthropology, University of Central Florida, Orlando, FL USA; 2 Indiana University Robert H. McKinney School of Law, Indianapolis, IN USA; 3 Department of Imaging and Pathology, Biomedical MRI Unit/Molecular Small Animal Imaging Center (MoSAIC), KU Leuven, Flanders, Belgium; 4 GREAB-Research Group in Biological Anthropology, Department of Evolutionary Biology, Ecology and Environmental Sciences (BEECA), Universitat de Barcelona (UB), Barcelona, Spain; 5 Center for Genomic Regulation (CRG), The Barcelona Institute of Science and Technology, Barcelona, Spain; 6 Universitat Pompeu Fabra (UPF), Barcelona, Spain; 7 CIBER Rare Diseases-CIBERER, Barcelona, Spain; 8 GTM-Grup de Recerca en Tecnologies Mèdia, Universitat Ramon Llull, La Salle, Barcelona, Spain; 9 Institució Catalana de Recerca I Estudis Avançats (ICREA), Barcelona, Spain; 10 EMBL Barcelona, European Molecular Biology Laboratory, Barcelona, Spain; 11 Integrative Pharmacology and Systems Neuroscience, IMIM-Hospital del Mar Medical Research Institute, Barcelona, Spain; 12 CIBER Physiopathology of Obesity and Nutrition-CIBERobn, Madrid, Spain

**Keywords:** Diseases, Translational research

## Abstract

Trisomy of human chromosome 21 (Down syndrome, DS) alters development of multiple organ systems, including the face and underlying skeleton. Besides causing stigmata, these facial dysmorphologies can impair vital functions such as hearing, breathing, mastication, and health. To investigate the therapeutic potential of green tea extracts containing epigallocatechin-3-gallate (GTE-EGCG) for alleviating facial dysmorphologies associated with DS, we performed an experimental study with continued pre- and postnatal treatment with two doses of GTE-EGCG supplementation in a mouse model of DS, and an observational study of children with DS whose parents administered EGCG as a green tea supplement. We evaluated the effect of high (100 mg/kg/day) or low doses (30 mg/kg/day) of GTE-EGCG, administered from embryonic day 9 to post-natal day 29, on the facial skeletal development in the Ts65Dn mouse model. In a cross-sectional observational study, we assessed the facial shape in DS and evaluated the effects of self-medication with green tea extracts in children from 0 to 18 years old. The main outcomes are 3D quantitative morphometric measures of the face, acquired either with micro-computed tomography (animal study) or photogrammetry (human study). The lowest experimentally tested GTE-EGCG dose improved the facial skeleton morphology in a mouse model of DS. In humans, GTE-EGCG supplementation was associated with reduced facial dysmorphology in children with DS when treatment was administered during the first 3 years of life. However, higher GTE-EGCG dosing disrupted normal development and increased facial dysmorphology in both trisomic and euploid mice. We conclude that GTE-EGCG modulates facial development with dose-dependent effects. Considering the potentially detrimental effects observed in mice, the therapeutic relevance of controlled GTE-EGCG administration towards reducing facial dysmorphology in young children with Down syndrome has yet to be confirmed by clinical studies.

## Introduction

Trisomy 21, an aneuploidy referred to as Down syndrome (OMIM 190685), is a complex genetic disorder and the most frequent genetic cause of intellectual disability, affecting one out of 700–1000 live-born individuals ^1^. The overexpression of hundreds of triplicated chromosome 21 genes causes a dosage imbalance that alters signaling pathways regulating the development of multiple tissues and organs ^1^ such as the brain, heart, immune system, gastrointestinal tract, and skeleton. Trisomy 21 is associated with characteristic facial features, including midfacial flatness, oblique palpebral fissures, mandibular hypoplasia, and facial asymmetry ^2^. Such dysmorphology, together with muscular dysfunction, result in a higher prevalence of malocclusion ^3^, mouth breathing and obstructive sleep apnea, and potentially compromises quality of life.

The genetic complexity of trisomy 21, combined with large degrees of developmental and phenotypic variability in individuals with Down syndrome, represents a serious challenge to any attempt to alleviate the alterations induced by this aneuploidy. Nevertheless, green tea extracts (GTE) enriched in catechins such as epigallocatechin-3-gallate (EGCG) have emerged as a promising therapeutic tool for individuals with Down syndrome ^4–9^. Although the effect of GTE-EGCG is not specific, and different catechins or combinations of catechins may have different effects on Down syndrome phenotypes ^7^, accumulating evidence suggests that the ability of GTE-EGCG to inhibit the excess kinase activity ^10^ of the dual specificity tyrosine-phosphorylation-regulated kinase 1A (*DYRK1A*) may play a prominent role ^11^.

The gene encoding for this kinase, *DYRK1A,* maps to the 21q22.2 region, whose duplication is associated with facial and cognitive phenotypes ^12^. *DYRK1A* is part of a complex genetic regulatory circuit at the crossroads of many important processes and developmental pathways, including development of the brain ^13^, face ^14^, skeleton ^6,7^, and heart ^15^. Thus, DYRK1A kinase inhibition by GTE-EGCG has the potential to influence multiple altered phenotypes associated with Down syndrome. Whether the effect of GTE-EGCG is through modulation of DYRK1A kinase activity or by acting on other genes or mechanisms is not completely characterized ^16^. However, it is broadly recognized that DYRK1A is a good therapeutic target for Down syndrome ^9,17^. Gene dosage imbalances in *DYRK1A* are associated with neural dysfunction, skeletal alterations, speech impairment, and/or brain and craniofacial dysmorphology ^18^ (i.e. microcephaly, brachycephaly). These phenotypic changes arise either from *DYRK1A* deficiency, such as in MRD7 autism spectrum disorder ^11^ and in syndromes caused by *DYRK1A* microdeletions ^19^, or from *DYRK1A* excess combined with other triplicated chromosome 21 genes, such as in Down syndrome ^20^.

Besides Dyrk1A kinase activity, GTE-EGCG may inhibit several *DYRK1A* related pathways such as the NFAT (Nuclear factor of activated T-cells) signaling pathway and transcription factors DCAF7 ^21^ and GLI1 ^22^, which are overexpressed in Down syndrome and play a role as critical regulators of vertebrate development and organogenesis, including skeletal, muscular and cardiac development ^15^. Specific to facial morphogenesis, GTE-EGCG could alter the role of *DYRK1A* in a network of calcium signaling genes regulated by retinoic acid (RA)^23^. Inhibiting transcriptional activity of NFAT by phosphorylation, DYRK1A can inactivate the transcription of genes that regulate neural crest cell migration and patterning of facial prominences during early orofacial development ^23^. Experimental research supports that *Dyrk1a* is expressed in the embryonic structures that give rise to the orofacial region and the brain ^14,23,24^, and contributes to regulate pre- and postanatal bone development ^25^. Thus, *DYRK1A* may have a direct role in facial development regulating the development of facial primordia into the osseous structures of the adult face, as well as an indirect role by controlling the growth of the forebrain ^24^, which acts as a mechanical substrate and a signaling center for early facial development ^26^. Through these complex signaling pathway interactions, *DYRK1A* may integrate craniofacial and brain development.

Overexpressed *DYRK1A* is also linked to cognitive impairment ^26^. Experimental mouse studies and clinical trials with adult individuals suggest that DYRK1A kinase inhibition by GTE-EGCG is involved in the improvement of adaptive functionality and cognitive deficits associated with Down syndrome ^5,8^. Studies using partially trisomic Down syndrome mouse models or transgenic mice that overexpress *Dyrk1a* indicate that GTE-EGCG ameliorates some cognitive ^26^, skull ^18^ and skeletal alterations ^6,7^. However, GTE-EGCG supplementation outcomes differ greatly depending on the treatment conditions (i.e. dosage, onset, duration of the treatment, as well as GTE composition, brand and administration method) ^27^. For example, a study in Ts65Dn mice found that 1 month of 2–3 mg of EGCG per day can improve cognition in adult mice ^5^, while another study failed to find such improvements in Ts65Dn mice treated with ~ 10, ~ 20, or ~ 50 mg/kg/day of EGCG starting at P24 for 3- or 7-weeks ^27^. Regarding skeletal development, EGCG administered to pregnant Ts65Dn dams (200 mg/kg/day on E7 and E8), permanently rescued cranial vault morphology in the offspring ^18^. Similarly, in 3 week old Ts65Dn mice, treatment with a low dose of EGCG (~ 9 mg/kg/day for 3 weeks beginning in early adulthood) improved femoral bone mineral density and trabecular microarchitecture ^6^. However, Ts65Dn mice treated with a higher dose ~ 50 mg/kg/day of EGCG for 7 weeks starting at P24 exhibited detrimental effects on femoral bone structure, such as reductions in cortical area, thickness, and structural strength ^27^. Consequently, an optimal effective GTE-EGCG treatment regime to improve cognitive development and morphogenesis in individuals with Down syndrome is still under debate ^27^.

To evaluate the therapeutic potential of GTE-EGCG upon facial development, we assessed the effects of two different doses of continued pre- and postnatal GTE-EGCG treatment on a mouse model of Down syndrome. We also analyzed, for the first time, facial morphology of children with Down syndrome that received different over-the-counter treatments based on GTE-EGCG supplementation.

## Results

### Dose-dependent effects of GTE-EGCG on facial shape in Down syndrome mouse model

We experimentally evaluated the effect of a green tea extract (GTE-EGCG; Mega Green Tea Extract, Life Extension, USA) on the facial skeletal development of a Ts65Dn (TS) mouse model. Ts(17^16^)65Dn mice carry a freely segregating marker chromosome that consists of a translocation of the telomeric region of Mmu16 onto the centromeric region on Mmu17, resulting in mice that are trisomic for orthologs of approximately half of the genes on Hsa21 ^28–30^. This well-established genetic murine model of Down syndrome reproduces many anatomical abnormalities found in humans with trisomy 21, including neuroanatomical changes, impaired learning, memory and craniofacial phenotypes ^31^. We administered either high (100 mg/kg/day) or low doses (30 mg/kg/day) of GTE-EGCG in drinking water to TS and wild-type euploid (WT) mice from embryonic (E) day 9 to post-natal (P) day 29 (see Table 1 for sample composition). The GTE-EGCG solution was prepared freshly using the same batch of the Mega Green Tea Extract in all the experiments, with an estimated composition of 53.6% EGCG, 4.5% epicatechin (EC), 9% epicatechin gallate (ECG) and 12.5% epigallocatechin (EGC). Control litters received a control solution (see “Materials and methods” for further details).

**Table 1:** Mouse sample composition by experiment of low and high dose GTE-EGCG treatment.

Treatment	WT	WT + GTE-EGCG	TS	TS + GTE-EGCG
Low dose	14 (7/7)	15 (4/11)	6 (4/2)	5 (3/2)
High dose		7 (3/4)		8 (4/4)

Sample sizes of treated and untreated wildtype (WT) and trisomic (TS) mice are provided, showing total number of mice, as well as number of mice separated by sex (Female/Male). Since permutation tests based on Procrustes distances between male and female mice showed that there are no significant facial shape differences due to sex (*P* = 0.2586) and male and female mice completely overlapped on the morphospace defined by a Principal Component Analysis (Fig. S2), we analysed the pooled data to increase the sample sizes and the robustness of the analyses.

**Figure 1:**
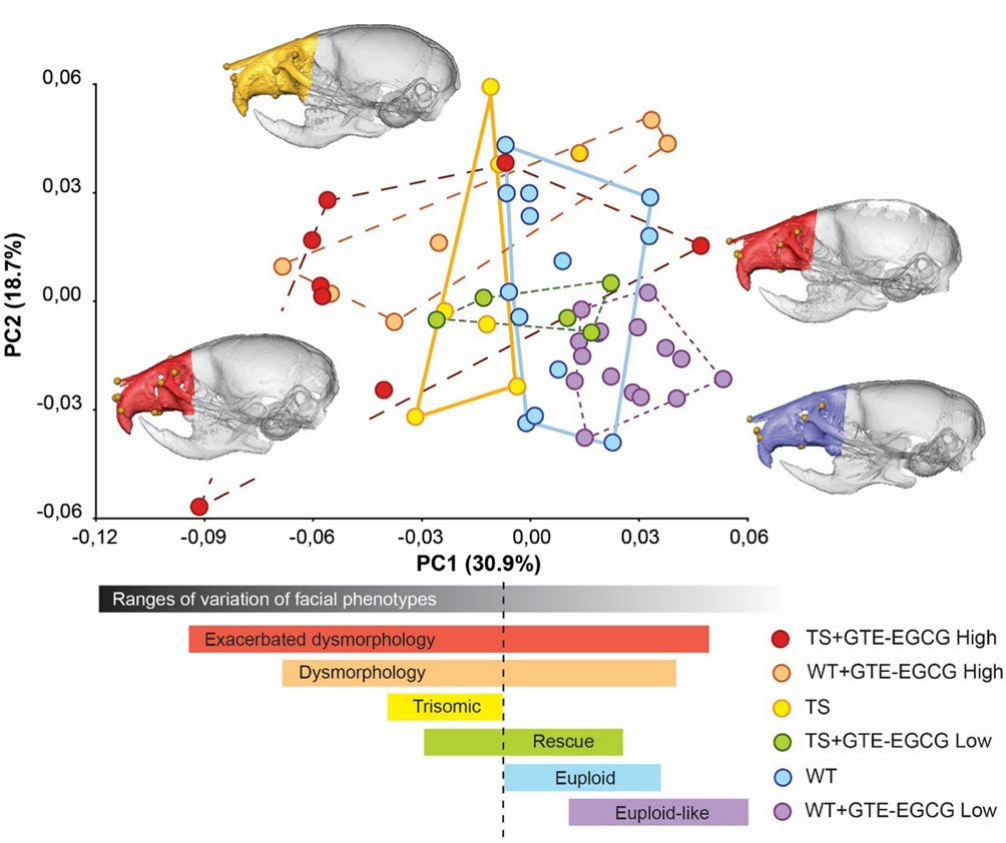
Principal Component Analyses of the global facial shape variation in Ts65Dn trisomic (TS) mouse models treated with high and low GTE-EGCG doses and euploid wild-type (WT) mice. Scatterplot of PC1 and PC2 axes with the corresponding percentage of total morphological variation explained is displayed along each axis. Convex hulls and horizontal color bars represent the ranges of variation within each group of mice. To visualize facial shape changes associated with positive and negative extremes of PC1 and PC2, skull CT reconstructions of mice occupying these positions are displayed. Note that although the complete skull is displayed for anatomical reference, only landmarks located on the colored facial region have been used for the analysis. Colors correspond to mice groupings. The red colored skull to the far right representing a TS mouse treated with a high dose of GTE-EGCG exhibited poor bone mineral density as evidenced by the nearly transparent regions of cranial vault bones. See Fig. S3 to further visualize facial shape changes associated to PC1, which is the axis that explains most morphological variation and separates groups based on genotype. Mice skull reconstructions were obtained from NRecon and Amira, and landmarking was performed with Amira.

We quantitatively compared the facial skeletal morphology of TS and WT mice, with and without treatment, using a quantitative shape analysis ^32^ based on the 3D coordinates of 12 anatomical facial landmarks registered on micro-computed tomographic (µCT) images of mouse craniofacial skeletons (Fig. S1A, Table S2). As sexual dimorphism was not a significant effect (Table 1 and Fig. S2), we performed our analyses on the pooled sample of male and female mice. To assess genotype and GTE-EGCG treatment effects on the murine facial skeleton, we performed a Principal Component Analysis (PCA). We explored facial shape variation among individuals by creating a morphospace based on the first two PCs, which together explained 49.6% of the total morphological variance among samples. In this scatterplot (Fig. 1), each point represented the facial shape of one individual and the experimental groups of mice were outlined using convex hulls. Close proximity between groups indicated similar facial phenotypes, while separation indicated dissimilarity. Our results showed genotype-dependent facial shape differences (Fig. 1). Untreated WT and TS mice clustered in separate morphospace groups with commensurate ranges of variation (Fig. 1). Compared to WT, TS mice exhibited midfacial hypoplasia and reduced facial dimensions, which corresponds to human trisomy 21 phenotypes (Fig. S3).

The PCA analysis also revealed a GTE-EGCG dose-dependent treatment effect. Mice treated with high GTE-EGCG doses exhibited atypically large facial morphological variation. Both WT and TS treated mice presented phenotypes that ranged from euploid to highly dysmorphic (Fig. 1), indicating that the effect of the high dose treatment was not homogeneous across groups and produced disparate effects. In the wild-type group, 43% of the WT treated mice fell close to the range of variation of non-treated WT mice and displayed typical euploid facial phenotypes, whereas 57% of the WT treated mice presented unusual facial dysmorphologies. In the trisomic group, 25% of the TS mice treated with high GTE-EGCG doses of the mice fell within the range of variation of WT non-treated mice, suggesting that the GTE-EGCG rescued the facial phenotype in those mice. However, the remaining 75% of the TS mice treated with the high dose occupied extreme morphospace positions associated with exacerbated facial dysmorphologies relative to non-treated TS mice (Fig. 1 and Fig. S3).

In contrast, low dose GTE-EGCG treatment produced more consistent effects across groups. Wild-type mice treated with low doses of GTE-EGCG presented euploid-like facial phenotypes within a range of variation that did not completely overlap with the range of variation of non-treated WT mice (Fig. 1). The GTE-EGCG treatment similarly affected all WT mice, deviating their facial morphology towards slightly longer snouts. In trisomic mice treated with a low dose of GTE-EGCG, the treatment did not have any apparent effect in 40% of the TS mice, as these animals fell within the range of variation of non-treated trisomic mice. However, the remaining 60% of TS mice exhibited facial phenotypes that overlapped with untreated WT mice (Fig. 1 and Fig. S3), suggesting that the low dose GTE-EGCG treatment rescued their facial dysmorphology.

Our results showed that the effect of high GTE-EGCG doses on facial shape was highly variable, unpredictable, and potentially detrimental for both TS and WT mice, indicating a deleterious effect of the high dose GTE-EGCG treatment on facial morphology. On the contrary, low doses of GTE-EGCG normalized facial development and shape in most TS mice.

**Figure 2:**
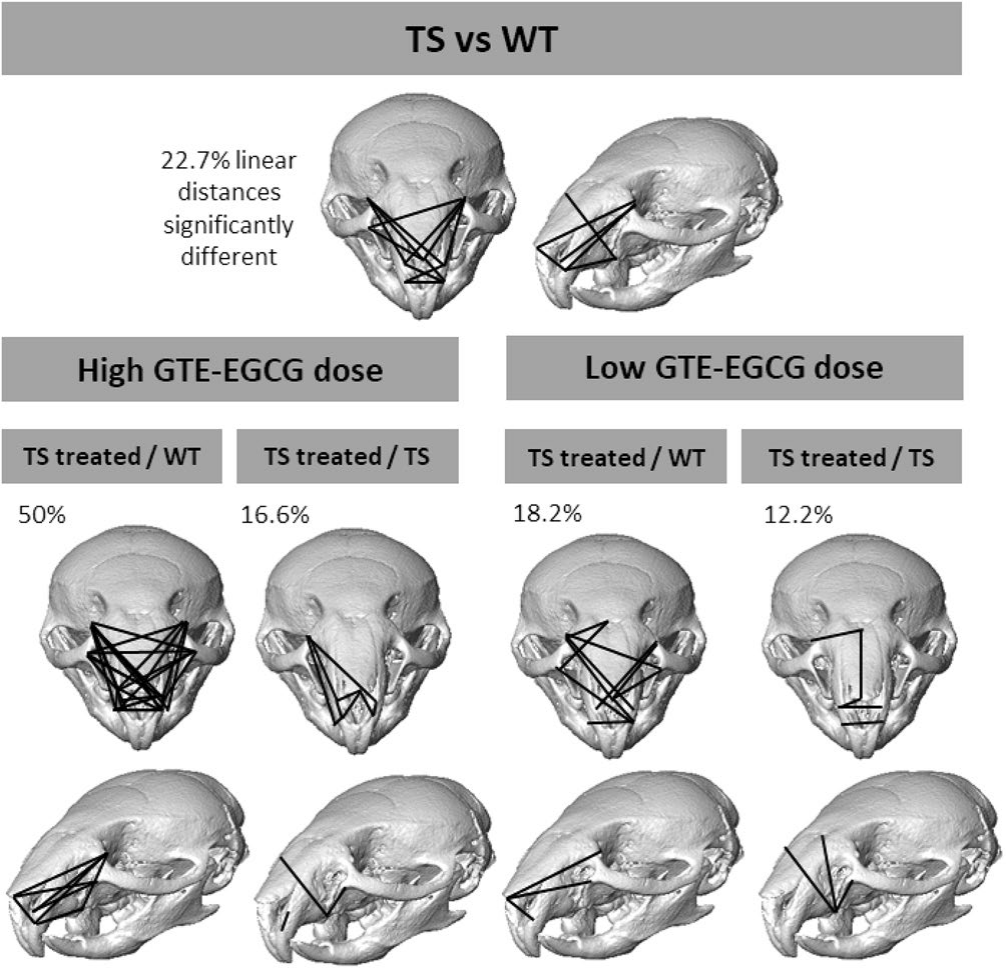
Localized Euclidean Distance Matrix Analysis facial shape pairwise contrasts for each mouse group of untreated wildtype (WT) and trisomic (TS) mice and low and high dose GTE-EGCG-treated mice. Results from the baseline contrast (TS vs WT) can be compared to results from high and low GTE-EGCG dose experiment to assess the effect of the treatment on Ts65Dn facial skeleton development. Black solid lines represent linear facial measurements that are significantly different in the two compared groups. Numbers indicate the percentage of significantly different linear distances within each comparison. Mice skull reconstructions were obtained from NRecon and Amira.

Finally, we carried out additional tests to evaluate the localized effects of GTE-EGCG treatment on facial shape. Euclidean Distance Matrix Analyses (EDMA) ^33^ revealed that 22.7% of facial measurements were significantly different between untreated TS and WT mice (Fig. 2). This percentage decreased to 18.2% when TS mice were treated with a low-dose of GTE-EGCG, suggesting a slight facial morphology improvement. In high-dose GTE-EGCG treated TS mice, the percentage of facial differences increased to 50% on average, confirming that facial dysmorphology dramatically increased in most trisomic mice when treated with the high dose (Fig. 2).

We also estimated facial treatment scores (FTS) by combining the EDMA results with an additional iterative bootstrapping method. This is a novel approach to statistically quantify whether a treatment improves a disease-altered morphology by reducing the morphological differences between treated and control groups. For each GTE-EGCG dose (high and low), FTSs were calculated as the relative difference in the percentage of significantly different facial traits between untreated and treated TS and WT mice (see “Materials and methods” for further details). We then implemented an iterative bootstrapping simulation approach to test FTS statistical significance. FTS values obtained from the experimental data were compared to randomly generated FTSs. For each high and low GTE-EGCG dose, we used an iterative scaffolding approach to generate multiple random pseudo-subsamples of WT and TS individuals that progressively increased the number of treated individuals in the trisomic sample (from 0 to 7 in the high dose experiment; and from 0 to 4 in the low dose experiment). In each iteration, we recomputed the EDMA results and FTS scores and compared the distribution of randomly generated FTS values with the experimental value obtained from the complete sample of treated individuals (eight in the high dose experiment; and five in the low dose experiment) (Fig. S4). Although neither test reached statistical significance, histograms showed FTS trends for each experiment that were consistent with previous results. In the high GTE-EGCG dose treatment comparison, the FTS decreased, indicating a worsening of facial shape. Conversely, in the low GTE-EGCG dose treatment comparison, the FTS increased, representing an improvement in facial shape. These results confirm a negative effect of high dose GTE-EGCG treatment, which on average increases facial differences between WT and TS mice, and a positive rescuing effect of low dose GTE-EGCG treatment on TS mouse model facial development.

### Reduced facial dysmorphology in children with Down syndrome taking early GTE-EGCG supplementation

We conducted a cross-sectional observational study to assess the facial shape in Down syndrome and to evaluate the effects of self-medication with green tea extracts in children from 0 to 18 years old (see Table 2 for cohort details and Table S1 for GTE-EGCG treatment details). The cohort included complete trisomy 21 cases who had or had not received GTE-EGCG supplementation, mosaic Down syndrome cases, and euploid children. In the GTE-EGCG treated group of children with Down syndrome, parents had administered their children commercially available green tea extracts containing EGCG obtained over-the-counter, for variable periods and at different doses. Thus, the study participants did not follow a systematic GTE-EGCG usage pattern, as the dosage, onset, and treatment duration were not standardized, and no euploid treated control group was available. However, this observational study assesses for the first time whether GTE-EGCG can alleviate facial dysmorphology in children with Down syndrome and reveal the need for a controlled clinical assay.

**Table 2:** Human cohort by age group.

	DS	Mosaic	GTE-EGCG	Euploid	Total
0–3 years	20 (8/12)	3 (2/1)	7 (3/4)	57 (34/23)	87 (47/40)
4–12 years	27 (14/13)	1 (1/0)	2 (0/2)	100 (54/46)	130 (69/61)
13–18 years	16 (8/8)	0	4 (0/4)	50 (30/20)	70 (38/32)
Total	63 (30/33)	4 (3/1)	13 (3/10)	207 (118/89)	287 (154/133)

Total number of children as well as separated numbers by gender (Female/Male) are provided. As sexual dimorphism may affect facial shape, we assessed facial differences between males and females by computing the Procrustes distances among gender groups and a permutation test (10,000 rounds) in each age category. 0–3 years old group: Sexual dimorphism was not a significant factor (*P*_(0–3)_ = 0.4965), and results did not differ when genders were analysed by separated (Figs. S3 and S4). 4–12 years old group: sexual dimorphism was not significant (*P*_(4–12)_ = 0.1470). However, as the sample size of the GTE-EGCG treated cases was limited, results are presented as “Supplementary Information” (Fig. S8). 13–18 years old group: despite sexual dimorphism was significant (*P*_(13–18)_ = 0.0138), we pooled male and female samples after checking that gender was not the main source of variation in the sample (Fig. S5) and that results separated by gender were consistent with the results from the pooled analyses (Fig. S6). In this group, we could not assess whether the treatment had a differential gender effect, as all the GTE-EGCG treated children were male.

We assessed global facial shape differences between children with Down syndrome and euploid controls using a Procrustes shape analysis based on the 3D coordinates of 21 anatomical facial landmarks identified on their 3D topographic images (Fig. S1B, Table S2). To determine whether the effects of GTE-EGCG supplementation were different depending on the developmental period, children were stratified into three developmental groups: babies/toddlers (0–3 years old), young children (4–12 years old), and adolescents (13–18 years of age) (Table 2). As sexual dimorphism was not a significant effect (Table 2), nor was the main source of facial morphological variation (Fig. S5), male and female data were pooled in all the analyses to increase sample sizes. We confirmed that the results from the pooled analyses were consistent with those resulting from analyses separated by sex (Fig. S6).

**Figure 3:**
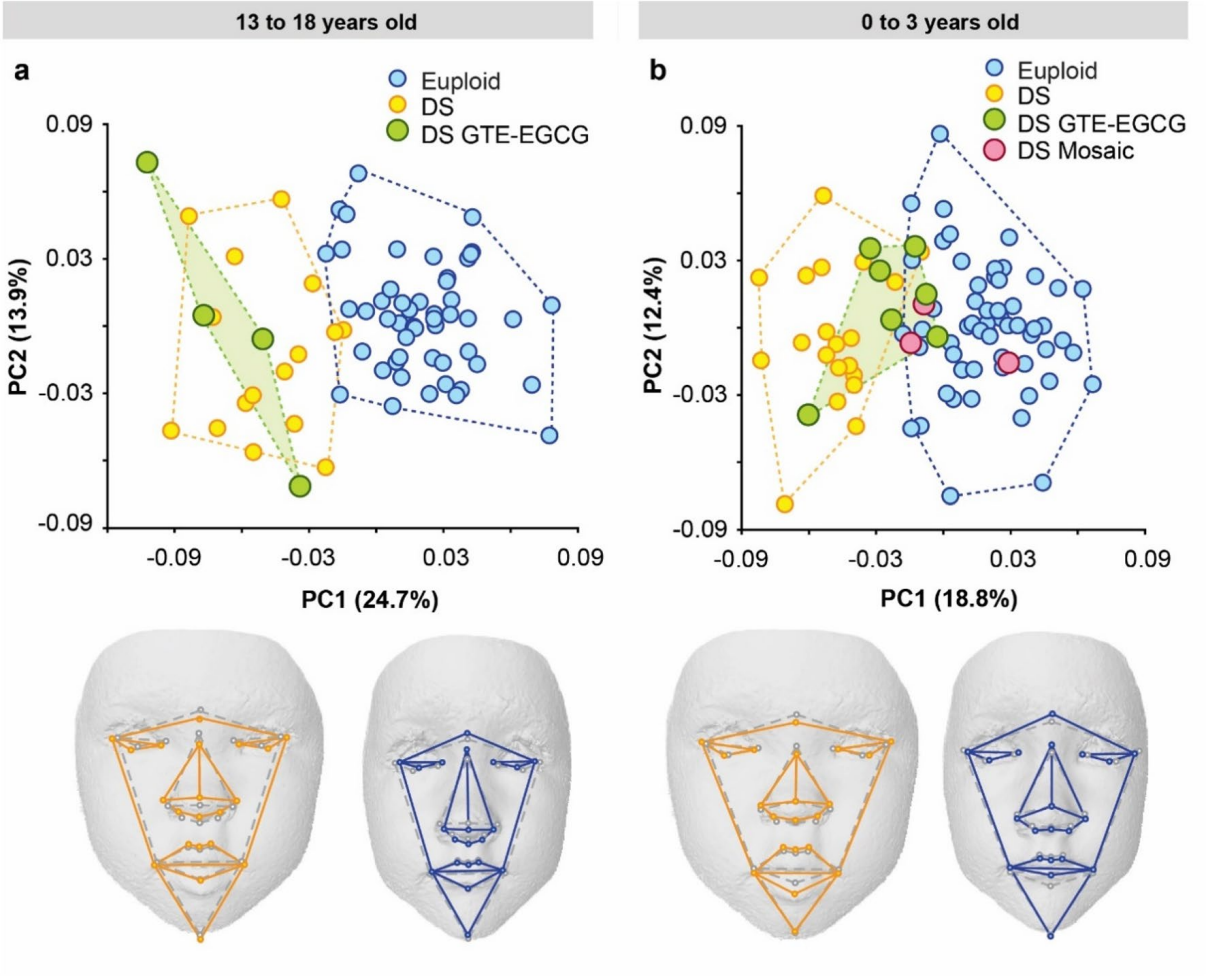
Principal Component Analyses of the global facial shape variation in human children from different age groups. (**a**) Adolescents from 13 to 18 years old, and (**b**) Babies/toddlers to 3 years old. Scatterplots of PC1 and PC2 axes with the corresponding percentage of total morphological variance explained are displayed along each axis. Convex hulls represent the ranges of variation within each group of children. Anterior facial morphings associated with the extreme negative and positive values of PC1 are also shown. Orange solid lines represents facial phenotypes associated with Down syndrome, whereas blue solid line represents facial phenotype associated with euploid condition. Both shapes are compared to average facial shape, represented by a dashed grey line. Facial reconstructions were obtained from 3dMD and Agisoft PhotoScan, landmarks were recorded with PhotoModeler and morphings performed with Amira.

Results showed genotype-dependent differences in facial shape (Fig. 3 and Figs. S7 and S8). Euploid and Down syndrome groups were variable, as shown by the dispersion of the data in the PC scatterplots (Fig. 3), but their ranges of variation did not overlap. Overall, individuals with complete trisomy 21 presented flat and wider faces, oblique palpebral fissures, flat nasal bridges, wider noses, and thicker lips in comparison to euploid faces, while those with mosaic Down syndrome exhibited intermediate phenotypes (Figs. 3 and S7).

The position of GTE-EGCG-treated children with Down syndrome in the morphospace was different depending on the age (Fig. 3). When treatment started late in adolescence, treated and untreated adolescents with Down syndrome completely overlapped in the morphospace (Fig. 3a), suggesting that the GTE-EGCG treatment conditions analyzed in this observational study did not show any relevant effect on facial shape during adolescence (Fig. 3a). At 4–12 years, GTE-EGCG treated children with Down syndrome tended to present intermediate phenotypes similar to mosaic cases, but larger samples are needed to confirm this result (Fig. S8). In the baby/toddler group, most children with Down syndrome supplemented with GTE-EGCG presented less severe facial phenotype as compared to untreated cases (Fig. 3b). In particular, six out of seven (85.7%) GTE-EGCG-treated children with Down syndrome showed less facial dysmorphology than untreated children with Down syndrome from the same age range. From these, three of the treated children fell within the euploid range of facial shape variation, similar to the toddlers with mosaic Down syndrome (Fig. 3b), while the other three treated children fell in an intermediate morphospace position between the ranges of variation of euploid and Down syndrome toddlers (Fig. 3b). This suggests that the beneficial effects of GTE-EGCG on facial dysmorphology are variable but can be detected in most of the treated cases.

**Figure 4:**
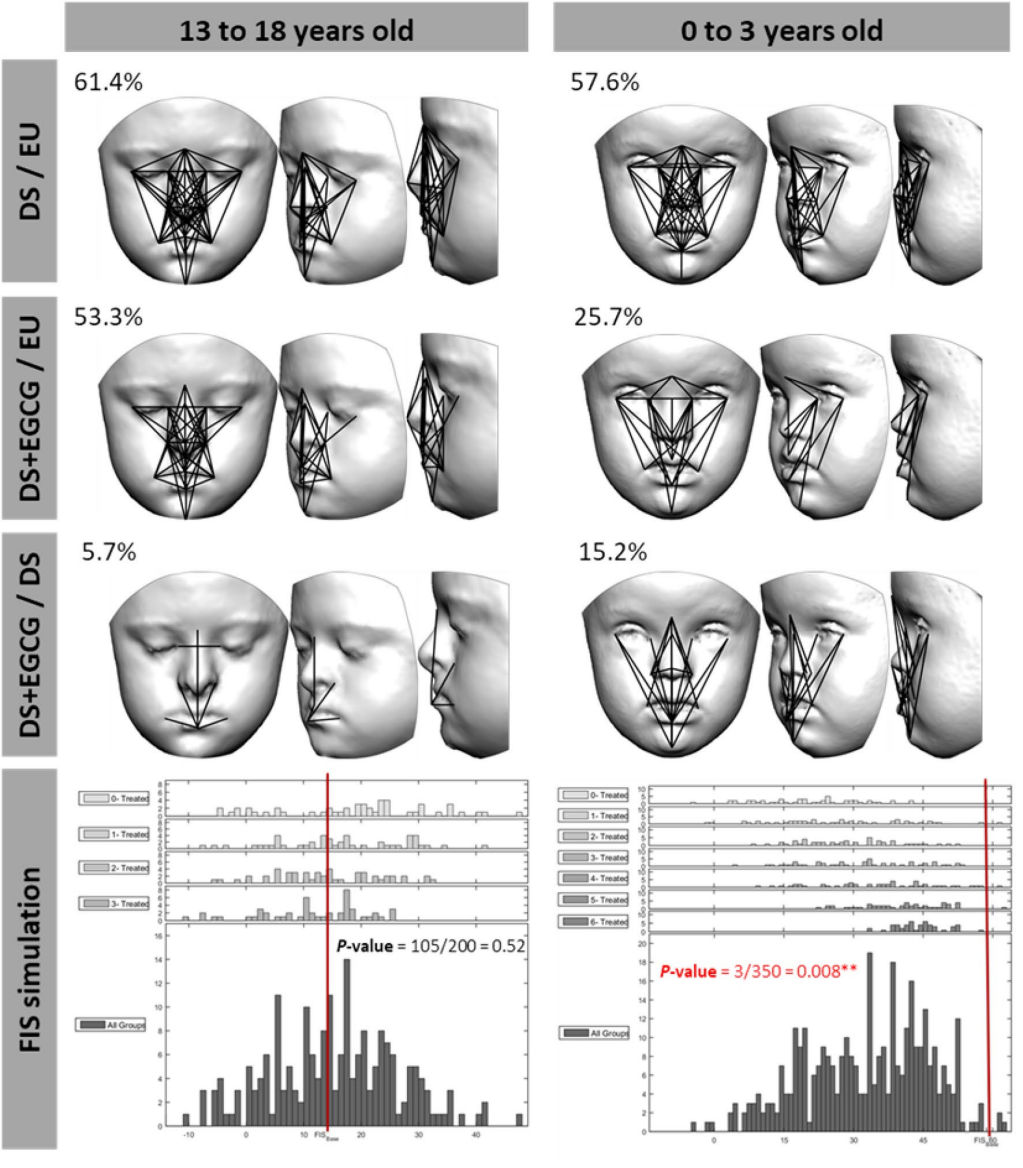
Localized Euclidean Distance Matrix Analysis facial shape pairwise contrasts and iterative bootstrapping tests of facial changes due to treatment for each age group. Black solid lines represent linear facial measurements that are significantly different in the two compared groups. First row: children with Down syndrome (DS) *versus* euploid children (EU); second row: children with Down syndrome supplemented with GTE-EGCG (DS + GTE-EGCG) *versus* euploid children (EU), third row: GTE-EGCG supplemented (DS + GTE-EGCG) *versus* untreated children with Down syndrome (DS), last row: iterative bootstrapping tests based on facial treatment scores (FTS). FTS is computed by contrasting the number of significant differences in facial traits between (1) euploid children and untreated children with Down syndrome and (2) euploid children and GTE-EGCG treated children with Down syndrome. Histograms represent the simulation results for each random group separately (top rows) as well as grouped together (bottom row). Each group contains an increasing number of GTE-EGCG treated Down syndrome cases. The red line shows the FTS score obtained with the complete set of observed cases, including all GTE-EGCG treated Down syndrome cases, in each group. *P*-values are provided for each group, and statistically significant comparisons are marked with **. Facial reconstructions were obtained from 3dMD and Agisoft PhotoScan.

To pinpoint the exact facial region on which GTE-EGCG may have an ameliorative effect, we analyzed local facial shape differences using EDMA at the different stages (Fig. 4). In the adolescent group, 61.4% of linear measures significantly differed between genotypes (Fig. 4). GTE-EGCG-treated adolescent cases showed a relatively low reduction of dysmorphology, with 53.3% of linear distance measures being still significant after treatment. Indeed, only a minimal number of significant differences were detected (5.7%) between untreated and treated adolescents with Down syndrome, supporting that GTE-EGCG has little to no effect on facial dysmorphology when supplementation is initiated during adolescence. The FTS estimated in this age group was low and failed to reach statistical significance (*P* = 0.52) (Fig. 4).

In babies and toddlers, GTE-EGCG supplementation was associated with changes in various dimensions of the nose, philtrum, and midface (Fig. 4). While 57.6% of linear distances were significantly different between euploid and untreated children with Down syndrome, in GTE-EGCG supplemented children only 25.7% of linear distances remained significantly different from euploids (Fig. 4). The FTS was high (58%) in this age group, indicating a large ameliorative effect of GTE-EGCG on facial shape (Fig. 4). In the simulation analyses, only three randomly simulated datasets of children with Down syndrome provided a higher FTS than the observational data (Fig. 4), indicating that the GTE-EGCG treatment effect was statically significant (*P* = 0.008).

Further analyses confirmed that the GTE-EGCG treatment significantly affects facial shape at early stages but not later in development in this observational study. We computed the Procrustes distances between euploid and Down syndrome treated and non-treated groups, and ran a permutation test for each age group. Results showed that when the treatment was taken during adolescence, facial shape did not significantly differ of non-treated adolescents with Down syndrome (*P* = 0.5248). In contrast, when the GTE-EGCG treatment was taken early in development, during the first years of life, its positive effect on facial shape was significant (*P* = 0.0348). A MANOVA test confirmed that at early ages there are no sex differences regarding the effect of the treatment on facial shape (*P* = 0.409), which indicates that GTE-EGCG treatment potential is the same for males and females.

## Discussion

Accumulating evidence is revealing that GTE-EGCG can modulate several aspects of phenotypes associated with trisomy 21 ^5–9,18^. GTE-EGCG is known to modulate bone metabolism ^34^, formation ^35^, and resorption ^36^, potentially by hindering osteoclastogenesis ^36^ and inducing osteoblast differentiation ^37^. Previous research has shown that high GTE-EGCG doses may suppress bone formation and exacerbate skeletal defects, whereas moderate GTE-EGCG doses may enhance bone formation and attenuate birth defects ^35,38^. Our study extends the evidence for the dosage dependency of the beneficial and detrimental effects of GTE-EGCG treatment to Down syndrome facial dysmorphology in Ts65Dn mice and provides the first observational evidence of supplementation effects in children with Down syndrome.

Our experimental results showed that GTE-EGCG treatment could reduce skeletal facial dysmorphology in trisomic Ts65Dn mice when administered from prenatal development until early adulthood at a low dose (30 mg/kg/day) (Figs. 1 and 2). However, the same treatment regime with a high GTE-EGCG dose (100 mg/kg/day) could have detrimental effects, exacerbating facial dysmorphology in some treated mice (Figs. 1, 2 and Figs. S3, S4). For instance, we observed that after low dose treatment, facial phenotype was rescued in some trisomic mice but not in others, whereas after high dose treatment, mice presented facial shape changes that ranged from relatively normal facial morphology to severe facial dysmorphology (Fig. 1). Further experimental research is needed to understand the mechanisms by which the same GTE-EGCG treatment can alter facial developmental and induce completely different facial phenotypes in mice with identical genotypes. We hypothesize that beyond a certain dosage, GTE-EGCG treatment can induce such divergent facial phenotypes by hindering developmental stability ^39^.

Since facial soft-tissues are built upon the bony skull scaffold and individuals with Down syndrome experience impaired bone formation and growth ^40^ (e.g., shortened limbs, low bone mineral content and density, reduced bone strength and remodeling, osteopenia and osteoporosis that worsens with age), we hypothesize that GTE-EGCG has a bone-remodeling effect on the face. Our results in the Ts65Dn mouse model support this assertion. Deficiencies in remodeling craniofacial bones ^41^ may explain the distinctive facial traits associated with trisomy 21. Midfacial hypoplasia, as well as the altered shape and position of the maxilla, mandible, nasal, and orbital cavities present in Down syndrome ^42^, may result from increased bone resorption, reduced bone deposition, and/or lack of proper growth adjustments of these craniofacial bones, all of which have a higher predisposition to resorption ^41^.

Our observational study suggests that GTE-EGCG supplementation may have beneficial effects on facial shape of children with Down syndrome at early age (Figs. 3 and 4). Despite all the variability present in the human sample regarding treatment, gender and age (Table S1), we detected significant differences in children with Down syndrome administered with low doses of GTE-EGCG during the first three years of life as compared to children with Down syndrome that never received the treatment (Figs. 3 and 4). These facial differences included length, width, and depth dimensions of the midface local to the sagittal plane, specifically on the philtrum and nasal regions (Fig. 4). In older age groups, however, we found no evidence of a GTE-EGCG supplementation effect on facial shape (Fig. 4). Since the expected effect of variability in non-controlled studies is to weaken the signal of the treatment effect, the variability of treatments included in this observational study may have underscored the effects of GTE-EGCG in facial morphology in children with Down syndrome (Figs. 3 and 4, and Fig. S8), rather than artificially strengthening them. Our results are thus preliminary and require confirmation by clinical trials to provide any conclusive evidence about GTE-EGCG treatment.

Therefore, we hypothesize that the potential of GTE-EGCG treatment in facial bone remodeling is highest during early development in children (Figs. 3 and 4), while the skull and the face exhibit high growth velocities and rates of development ^43^, driven by rapid brain expansion and masticatory functional changes as offspring are weaned to solid foods. Afterwards, facial growth is much slower and bone remodeling activity is reduced. When the face has attained its final size and shape, usually around 13–16 years of age ^43^, there is little possibility to modify the facial shape since bone remodeling occurs only at low rates for homeostatic maintenance.

In conclusion, our experimental results, supported by the observational data, suggest that GTE-EGCG can modulate facial development to reduce dysmorphogenesis in Down syndrome. We report for the first time that, besides cognition and the skeletal system, green tea extracts may have gene-dosage dependent effects on facial shape. Further preclinical and clinical research is needed to explain the discordant and pleiotropic effects of GTE-EGCG under different treatment conditions and to identify an ideal therapeutic window and treatment regime to reduce facial dysmorphologies and associated comorbidities in Down syndrome. These investigations will pinpoint optimal dosage and timing for GTE-EGCG treatment, minimizing side effects and maximizing cognitive and biological growth benefits.

This research is critical because GTE-EGCG is commercially accessible over-the-counter and we found potentially detrimental developmental effects on both trisomic and euploid conditions that warn against the generalized, non-prescribed intake of high GTE-EGCG doses as a health-promoting measure ^44^. To effectively translate this potential treatment into clinical practice, clinical trials with larger cohorts and controlled administration simultaneously assessing cognition, skeletal development, and growth on large pediatric samples are mandatory to identify the GTE-EGCG treatment that can reliably improve development in children with Down syndrome.

## Materials and methods

### Experimental design

We performed two controlled experiments using the Ts65Dn mouse model to assess the effect of high and low GTE-EGCG treatment doses on the shape of the facial skeleton of adult mice. The study was carried out in compliance with the ARRIVE guidelines (Animal Research: Reporting of In Vivo Experiments).

#### Breeding conditions

Mice were housed at the animal facility of KU Leuven in standard individually ventilated cages (40 cm long × 25 cm wide × 20 cm high) under a 12 h light/dark schedule in controlled environmental conditions of humidity (50–70%) and temperature (22 ± 2 °C) with food and water supplied ad libitum. Water intake was monitored by cage.

#### Animals

For each experiment, we established a breeding colony composed of 4 WT males and 6 Ts65Dn females (Refs. 001875 and 001924, the Jackson Laboratory Bar Harbor, ME, USA). The Ts65Dn is a widely studied DS mouse model ^45,46^ that carries a segment with approx. 120 genes homologous to Hsa21 (starting upstream of Mrpl39 to the telomeric end of Mmu16), translocated to a small centromeric part of Mmu17 that is not syntenic to Hsa21 ^28,30^. In total, we bred 55 Ts65Dn mice from eight litters (Table 1). Experimental protocols complied with all local, national and European regulations and were authorized by the Animal Ethics Committee of the KU Leuven (ECD approval number P004/2016) to optimize the minimization of animal sample size versus maximizing conclusive output in terms of statistical significance.

#### Treatment

We administered EGCG to 50% of randomly chosen pregnant dams to produce approximately the same number of GTE-EGCG-treated and non-treated mice. In one experiment, mice were treated with a high dose of GTE-EGCG (100 mg/kg/day), whereas in the other experiment mice received a lower dose (30 mg/kg/day). Since EGCG crosses the placenta and reaches the embryo ^4^, treatment was administered prenatally at embryonic day 9 (E9). We chose this time point to optimize treatment efficiency, because this is when the face starts developing and *Dyrk1A* begins to be expressed in mouse embryos. GTE-EGCG treatment continued pre- and postnatally over the duration of the experiment, from E9 until adulthood at P29. During gestation and lactation, GTE-EGCG was delivered to dams dissolved in water at a concentration of 326 mg/L in the high dose experiment and 90 mg/L in the low dose experiment. After weaning at postnatal day 21 (P21) and until P29, GTE-EGCG dissolved in water at the same concentrations was made available to young mice that drank ad libitum. The GTE-EGCG solution was prepared freshly from a green tea extract (Mega Green Tea Extract, Life Extension, USA; EGCG content of 45% or 326.25 mg per capsule) every 3 days. We monitored water intake to control the amount of EGCG consumed by the dams and weaned mice. The dosage received was thus approximately 100 mg/kg/day in the high dose experiment, and 30 mg/kg/day in the low dose experiment for an adult mouse, considering that, on average, early adult mice weigh 20 g and drink 6 ml of water per day.

#### Morphometric facial study

Mice were genotyped by quantitative PCR from ear snips and were distributed in four groups according to their genotype and treatment: Euploid (WT), euploid treated with GTE-EGCG (WT + GTE-EGCG), trisomic Ts65Dn (TS) and trisomic Ts65Dn treated with GTE-EGCG (TS + GTE-EGCG). See Table 1 for details on sample composition. We acquired in vivo whole-body micro computed tomography (µCT) scans of mice at P29 using a high resolution (50 μm isotropic) dedicated fast low-dose in vivo µCT scanner (SkyScan 1278, Bruker Micro-CT, Kontich, Belgium). Free-breathing mice were scanned under isoflurane anesthesia (~ 1–2% in oxygen), while physiological functions were constantly monitored and maintained (temperature, breathing, visual inspection via camera). Scans were reconstructed using NRecon software (v1.6.10.4), whereas skull visualization and landmarking were performed by a single observer using Amira software (v6.3, Visualization Sciences Group, FEI). Measurement error was within the range of previous studies ^47^. To capture the shape differences between WT and TS mice and to assess whether EGCG treatment had an effect on facial development, we recorded the three-dimensional coordinates of a set of 12 facial anatomical landmarks (Fig. S1A and Table S2).

### Patient cohort and recruitment

The human sample included 288 European and North American children from 0 to 18 years old recruited for photographic sessions at schools, research centers, Down syndrome OPTIONS and family congresses between 2009 and 2017 (Table 2 and Table S1). All protocols were approved by ethics committees: Comité Ético de Investigación Clínica-Parc de Salut MAR, Nº 2012/4849/I; modificación 24/07/2015; University Central Florida Institutional Review Board approval SBE-15-11524). To photograph the children and to obtain relevant clinical information, we obtained informed consent from their parents or legal guardians. All methods were carried out in accordance with relevant guidelines and regulations.

#### Facial image acquisition and anatomical landmark collection

Human faces were digitalized using non-invasive photogrammetry techniques. The facial images of each individual were acquired in an upright position with neutral facial expression using either a 3dMD Face system (3dMD, Atlanta, GA) or self-built photographic rigs, as described elsewhere ^42^. Each system acquired 5–10 images simultaneously of an individual’s face and used photogrammetry to merge separate two-dimensional (2D) images into a single three-dimensional (3D) model that accurately represents facial shape. The software used for obtaining the 3D reconstructions was 3dMD (3dMD, Atlanta, GA), PhotoModeler (v 1.1.1546, Eos Systems, Inc. Canada), and AgiSoft PhotoScan Standard (v 1.3.2). All data was collected with high precision and accuracy based on the same principles of stereo photogrammetry. Scale differences from the different acquisition systems were accounted for in the subsequent analyses by transforming pixel sizes to their corresponding mm measures.

We measured and recorded 3D coordinates of 21 facial landmarks from each individual’s 3D image following published protocols ^42^ (Fig. S1B and Table S2). Measurement error was within the range of previous studies (0.26 mm along the x-dimension, 0.30 mm along the y-dimension, and 0.31 mm along the z-dimension) ^2^ and thus considered negligible. Following inspection of landmark coordinates for gross error, anatomical landmark coordinates were used to conduct morphometric analyses.

#### GTE-EGCG supplementation

In this observational study, the cohort included cases of children with Down syndrome whose parents had administered them commercially available green tea extracts containing EGCG obtained over-the-counter. GTE-EGCG supplementation was variable for onset and duration of treatment, dosage and brand, as specified for each individual in Table S1.

### Statistical analysis

We compared facial shape by integrating results from different morphometric methods ^32,33^, Procrustes and Euclidean Distance Matrix Analysis (EDMA), using MorphoJ ^48^ and Matlab (R2018a, MathWorks, Inc., Natick, MA, USA).

#### General Procrustes Analysis (GPA)

We used this analysis ^32^ to assess global facial phenotype, analyzing facial shape as a whole 3D landmark configuration. Using this method, the 3D spatial relationship between landmarks was always preserved throughout the analysis. Following the standard General Procrustes Analysis, landmark facial shape configurations of all individuals were superimposed by shifting them to a common position, rotating, and scaling them to a standard size until a best fit of corresponding landmarks was achieved. Although the resulting Procrustes coordinates were scale-free, the shape data still contained a component of size-related shape variation due to allometry ^49^, which refers to organism shape change relative to body size changes that occur from growth during development. Since our human dataset included children of different ages, we removed the allometric effects by performing a multivariate regression of shape on age within each age group ^49^. The Procrustes residuals from this regression were the input for further statistical analysis.

#### Principal Component Analysis (PCA)

This multivariate technique was used to explore facial morphological variation ^32^ and to assess whether individuals grouped according to their genotype and/or treatment. PCA performs an orthogonal decomposition of the data and transforms variance–covariance matrices into a smaller number of uncorrelated variables called Principal Components (PCs), which successively account for the largest amount of variation in the data. Each individual is then scored for every PC, and individuals are plotted using these scores along the morphospace defined by the principal axes as an ordination method to visualize patterns of shape variation associated with individual axes. The distribution of individuals and groups throughout the morphospace along different PC axes reveals similarities or differences, based on proximity to others, in facial shape and variation among groups. This ordination method allowed us to assess whether individuals with Down syndrome treated with GTE-EGCG presented an intermediate or rescued facial phenotype relative to untreated individuals with Down syndrome and euploid controls along the PC axes explaining the most variance across groups.

#### Euclidean Distance Matrix Analysis (EDMA)

This is a robust morphometric method ^33^ for assessing local differences between samples because it pinpoints exactly which linear measurements significantly differ between pairwise sample contrasts and compares patterns of significant differences across samples. In the human sample, two-tailed two-sample shape contrasts were conducted on all unique inter-landmark linear distances from each sample using a non-parametric bootstrap (10,000 resamples). A total of 210 unique facial measurements were calculated for each individual from Procrustes-based allometrically corrected shape coordinates. Linear distances were compared for each age group (0–3 years, 4–12 years, 13–18 years) of Down syndrome, Down syndrome treated with GTE-EGCG, and euploid individuals. Euclidean Distance Matrix Analysis statistically evaluated the number of significant local linear distances in each two-sample comparison based on confidence interval testing (α = 0.10). Significant differences were plotted upon facial figures to pinpoint specific local shape differences and reveal the unique morphological patterns of variation associated with each pairwise contrast.

#### *Facial treatment *score

We assessed whether facial change due to GTE-EGCG treatment was statistically significant using a novel morphometric method that combines EDMA and iterative bootstrapping. First, we estimated relative facial change from the EDMA results as the following percentage: [% significant distances (Down syndrome vs euploid) − % significant distances (Down syndrome treated with GTE-EGCG vs euploid)/% significant distances (Down syndrome vs euploid)] × 100. To assess the statistical significance of the patterns revealed by EDMA, we ran iterative bootstrapping tests. We performed a subsampling approach in which we generated random pseudo-subsamples with increasing numbers of Down syndrome treated cases. For each age group, the whole sample was divided into groups of N individuals (where N is the number of GTE-EGCG-treated individuals with Down syndrome in the original sample) following a subsampling approach that randomly created pseudo sub-samples containing a known number (namely M) of treated individuals, resulting in a series of staggered pseudo sub-samples containing from M = 0 to M = N treated individuals. We computed an EDMA and a facial treatment score (FTS) corresponding to each simulation and analyzed the FTS histogram of each random group. Finally, we computed an aggregated FTS histogram of all groups and compared the distribution of random FTSs with the observed FTS. The *P*-value of the comparison was computed as the ratio between the number of simulations that provided a higher FTS than the observed FTS and the total number of simulations and was used to assess statistical significance (α = 0.05).

## Supplementary Information

Supplementary Information.

## Data Availability

Raw phenotype data cannot be made available due to restrictions imposed by the ethics approval. Because of data size, mice scan data is available upon simple reasonable request to the corresponding author. Anonymized landmark data and Matlab code for computing Facial treatment score (FTS) is available at https://github.com/xaviersevillano/FIS_Analysis_3D.
