# Supplementary material for: Green tea extracts containing epigallocatechin-3-gallate modulate facial development in Down syndrome
Source: Sci Rep. 2021 Feb 25;11:4715. doi: 10.1038/s41598-021-83757-1 (PMC7907288; doi:10.1038/s41598-021-83757-1)
Supplement: Supplementary file 1 — Supplementary Information. [file 41598_2021_83757_MOESM1_ESM.pdf]

SUPPLEMENTARY DATA

Full Title

Green Tea Extracts Containing Epigallocatechin-3-Gallate Modulate Facial Development in Down Syndrome

Short title

Facial Remodeling in Down Syndrome.

Authors

John M. Starbuck (PhD),[1,2] Sergi Llambrich (MSc),[3] Ruben González (MSc),[4] Julia Albaigès (MSc) ,[5,6,7] Anna Sarlé (MSc),[4] Jens Wouters (BSc),[3] Alejandro González (PhD),[8] Xavier Sevillano (PhD),[8] James Sharpe (PhD),[5,9,10] Rafael De La Torre (PharmD, PhD),[11,12] Mara Dierssen (MD, PhD),[5,6,7]† Greetje Vande Velde (BM, MScEng, PhD),[3]† Neus Martínez-Abadías (PhD)[4,5,10]*†

Affiliations

[1] Department of Anthropology, University of Central Florida, Orlando, FL, USA

[2] Indiana University Robert H. McKinney School of Law, Indianapolis, IN, USA

[3] Biomedical MRI unit/Molecular Small Animal Imaging Center (MoSAIC), Department of Imaging and Pathology, KU Leuven, Flanders, Belgium

[4] GREAB-Research Group in Biological Anthropology. Department of Evolutionary Biology, Ecology and Environmental Sciences, BEECA. Universitat de Barcelona, Barcelona, Spain

[5] Center for Genomic Regulation (CRG), The Barcelona Institute of Science and Technology, Barcelona, Spain

[6] Universitat Pompeu Fabra (UPF), Barcelona, Spain

[7] CIBER Rare Diseases–CIBERER, Barcelona, Spain

[8] GTM–Grup de Recerca en Tecnologies Mèdia, La Salle, Universitat Ramon Llull, Barcelona, Spain

[9] Institució Catalana de Recerca i Estudis Avançats (ICREA), Barcelona, Spain

[10] EMBL Barcelona, European Molecular Biology Laboratory, Barcelona, Spain

[11] Integrative Pharmacology and Systems Neuroscience, IMIM–Hospital del Mar Medical Research Institute, Barcelona, Spain

[12] CIBER Physiopathology of Obesity and Nutrition–CIBERobn, Madrid, Spain

* Corresponding author: Neus Martínez-Abadías, neusmartinez@ub.edu, Facultat de Biologia, Universitat de Barcelona (UB), Avda. Diagonal, 643. Planta 2. 08028 Barcelona (Spain)
† Shared last authorship

SUPPLEMENTARY DATA OVERVIEW

Supplementary figures overview:

Fig. S1. Set of anatomical landmarks used to characterize the facial morphology in adult mice (A) and children (B).
Fig. S2. Principal Component Analyses of the facial shape variation in mice.
Fig. S3. Facial shape changes in Down syndrome mouse models associated with PC1 axis, from negative to positive values.
Fig. S4. Iterative bootstrapping tests based on facial improvement scores (FIS) in Down syndrome mouse models that received an experimental prenatal GTE-EGCG treatment.
Fig. S5. Principal Component Analyses of the global facial shape variation in male and female children from old (13-18 years) and young (0-3 years) age groups.
Fig. S6. Principal Component Analyses of the global facial shape variation in children from different age groups and diagnostic, separated by sex.
Fig. S7. Facial shape changes in children with Down syndrome.
Fig. S8. Principal Component Analyses of the global facial shape variation in children from 4 to 12 years old.

Supplementary tables overview:

Table S1. Demographic and clinical details of all children with Down syndrome treated with GTE-EGCG in each age group.
Table S2. Definitions of facial landmarks in adult mice (A) and children (B).

SUPPLEMENTARY MATERIALS

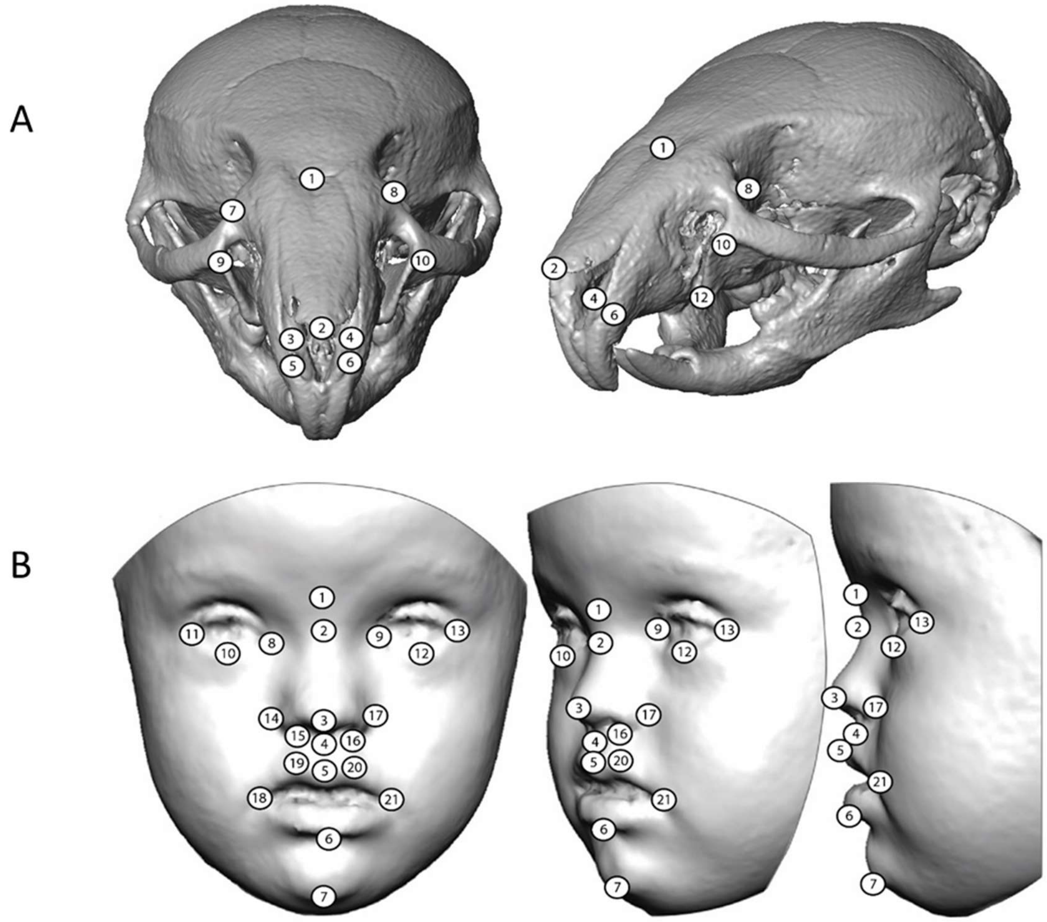

Fig. S1. Set of anatomical landmarks used to characterize the facial morphology in adult mice (A) and children (B). See Table S2 for precise anatomical definitions. Mice skull reconstructions obtained from NRecon and Amira, and landmarks recorded with Amira. Facial reconstructions obtained from 3dMD and Agisoft PhotoScan, and landmarks recorded with PhotoModeler and Amira.

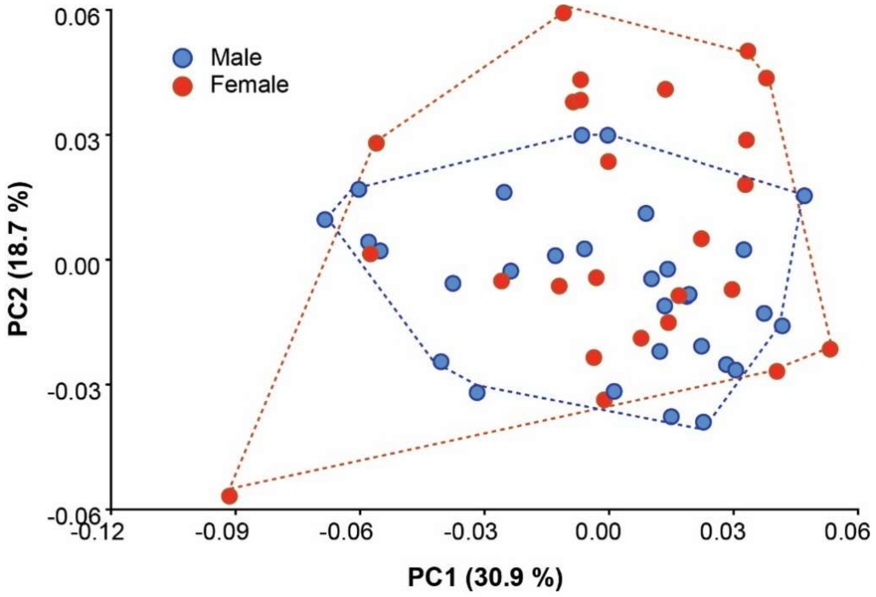

Fig. S2. Principal Component Analyses of the facial shape variation in mice. Scatterplots of PC1 and PC2 axes where convex hulls represent the ranges of variation within males (blue) and females (red). This sample is the same as the one used in Fig. 1.

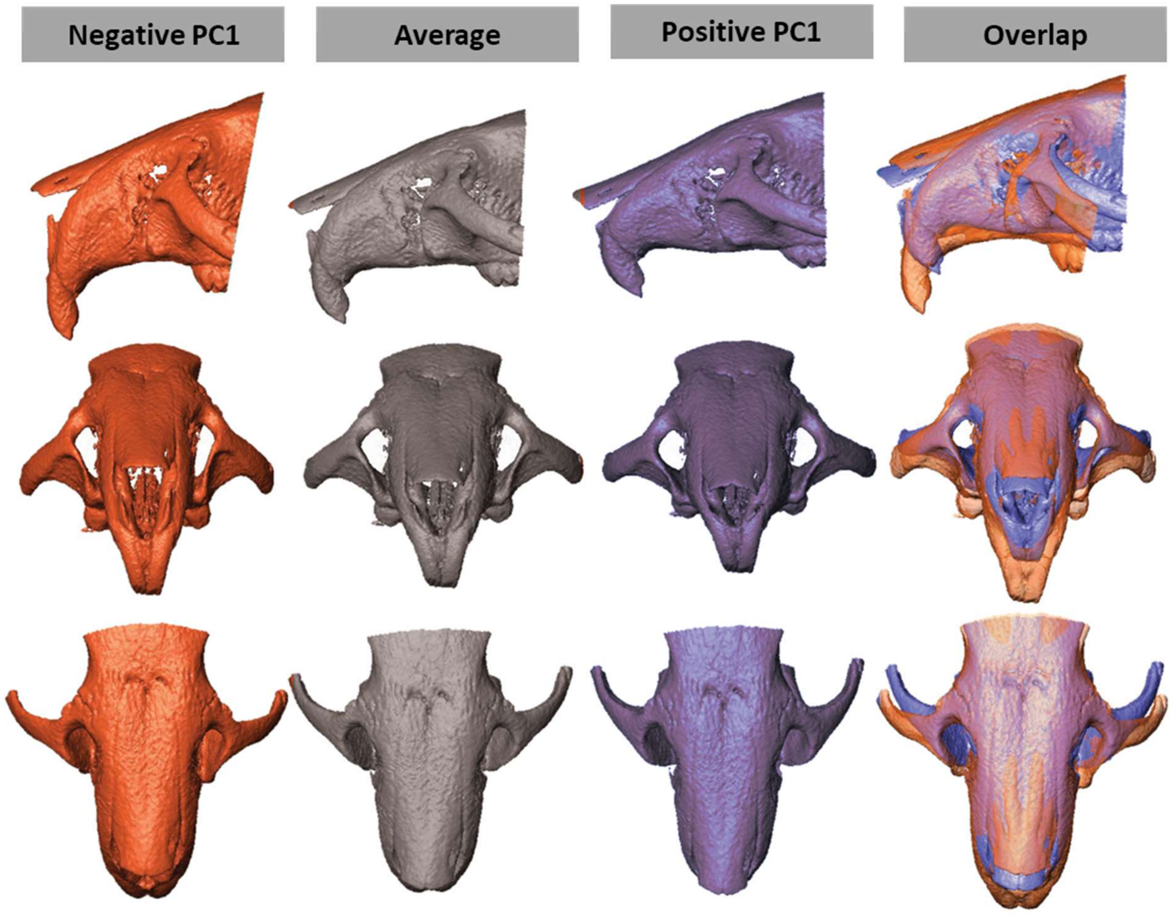

Fig. S3. Facial shape changes in Down syndrome mouse models associated with PC1 axis, from negative to positive values. 3D facial reconstructions from *in vivo* μ-CT scans are shown from a lateral view (top row), an anterior view (middle row), and superior view (bottom row). See Fig. 1 for distribution of mice along this axis. Mice facial reconstructions obtained from NRecon and Amira, and morphings performed with Amira.

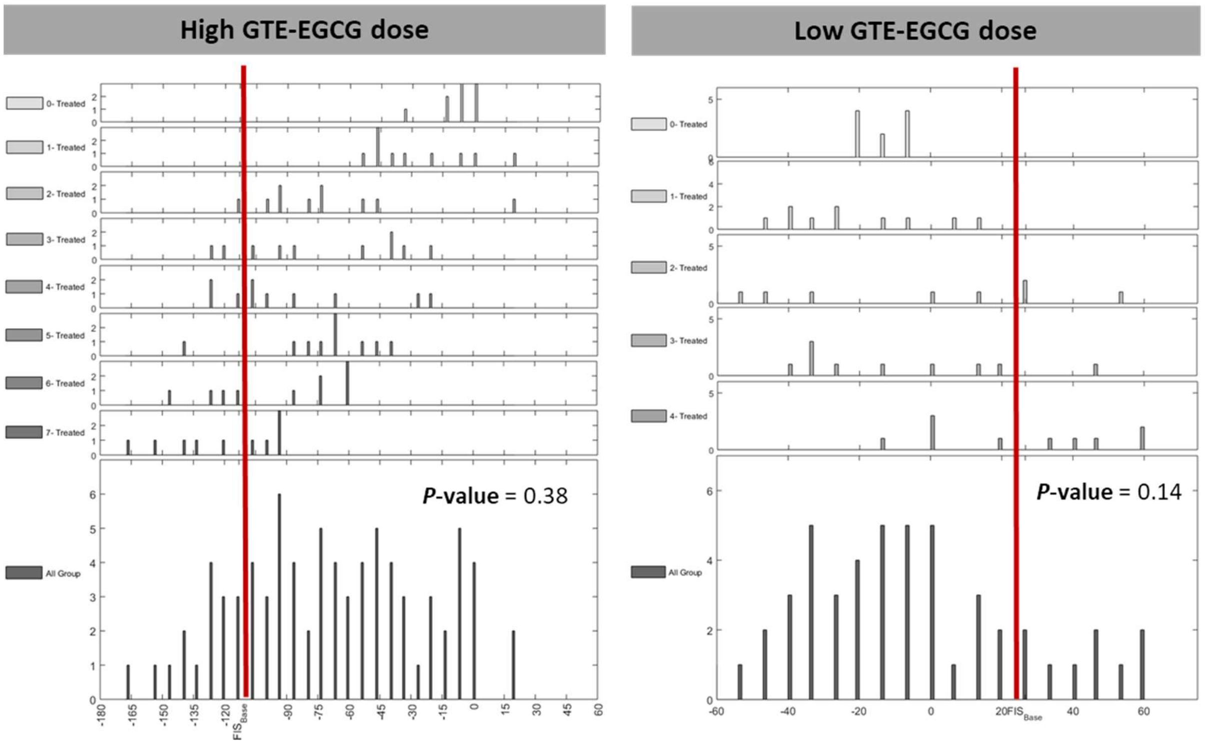

Fig. S4. Iterative bootstrapping tests based on facial improvement scores (FIS) in Down syndrome mouse models that received an experimental prenatal GTE-EGCG treatment. FIS is a summary value that was computed by contrasting the number of significant differences in facial traits between 1) WT mice and TS mice not treated with GTE-EGCG and 2) WT mice and TS mice treated with GTE-EGCG. Histograms representing the bootstrapping results for each random group are provided separately as well as aggregately. Each group contains an increasing number of GTE-EGCG treated mice. The red line shows the FIS score obtained with the observed GTE-EGCG treated mice. *P*-values are provided for each group.

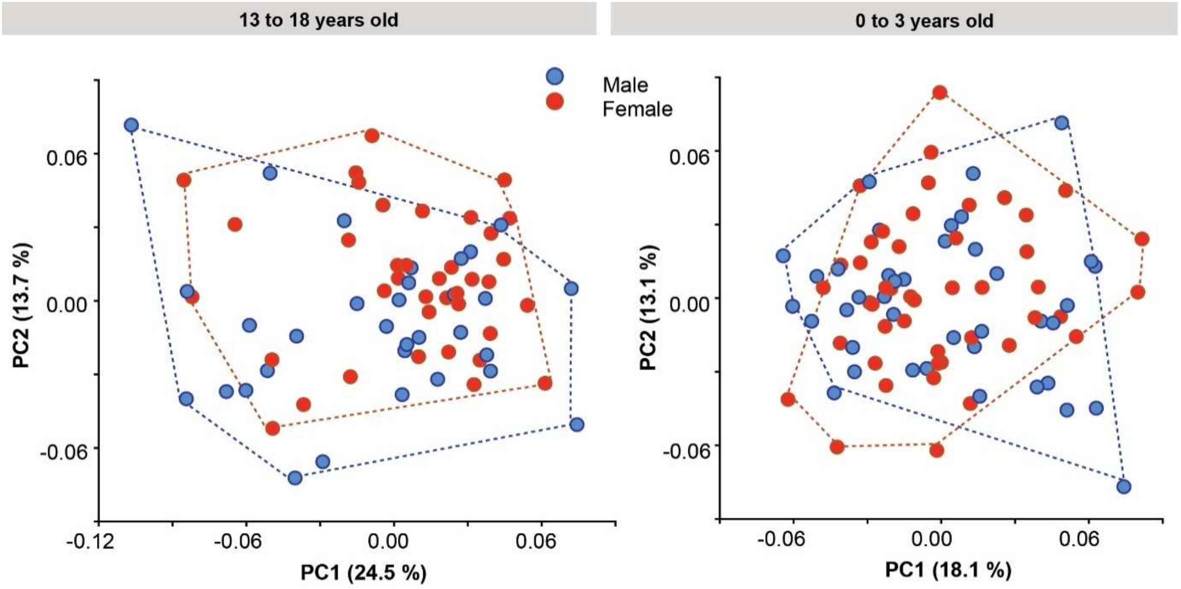

Fig. S5. Principal Component Analyses of the global facial shape variation in male and female children from old (13-18 yrs.) and young (0-3 yrs.) age groups. Scatterplots of PC1 and PC2 axes with convex hulls representing the ranges of variation within males (blue) and females (red). Results showed that male and female individuals completely overlapped in the morphospace and that the diagnostic condition is the main source of facial morphological in this sample, as shown in Fig. 3.

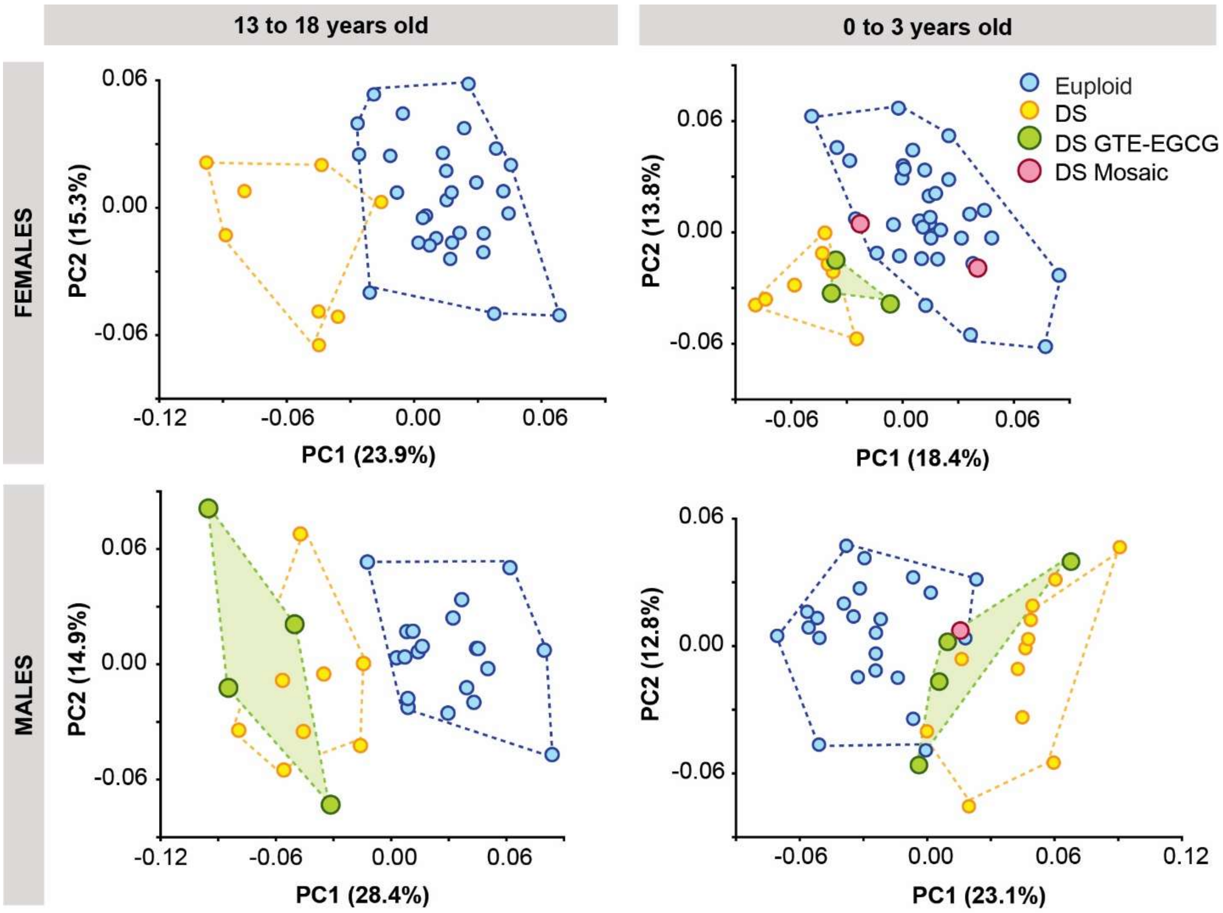

Fig. S6. Principal Component Analyses of the global facial shape variation in human children from different age groups and diagnostic, separated by sex. Scatterplots of PC1 and PC2 axes where convex hulls represent the ranges of variation within Down syndrome children (red), Down syndrome children treated with EGCG (green), euploid children (blue) and mosaic cases (purple).

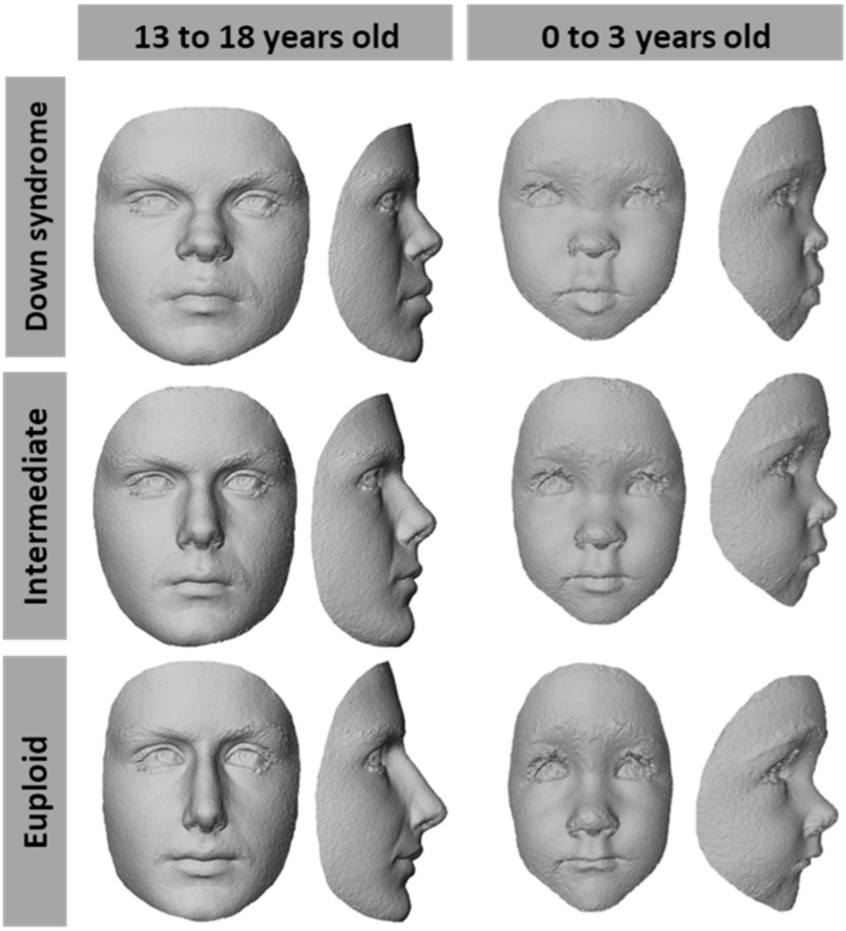

Fig. S7. Facial shape changes in children with Down syndrome. See Fig. 3 for distribution of children along the corresponding PC1 axis. Facial reconstructions obtained from 3dMD and Agisoft PhotoScan, and morphings performed with Amira.

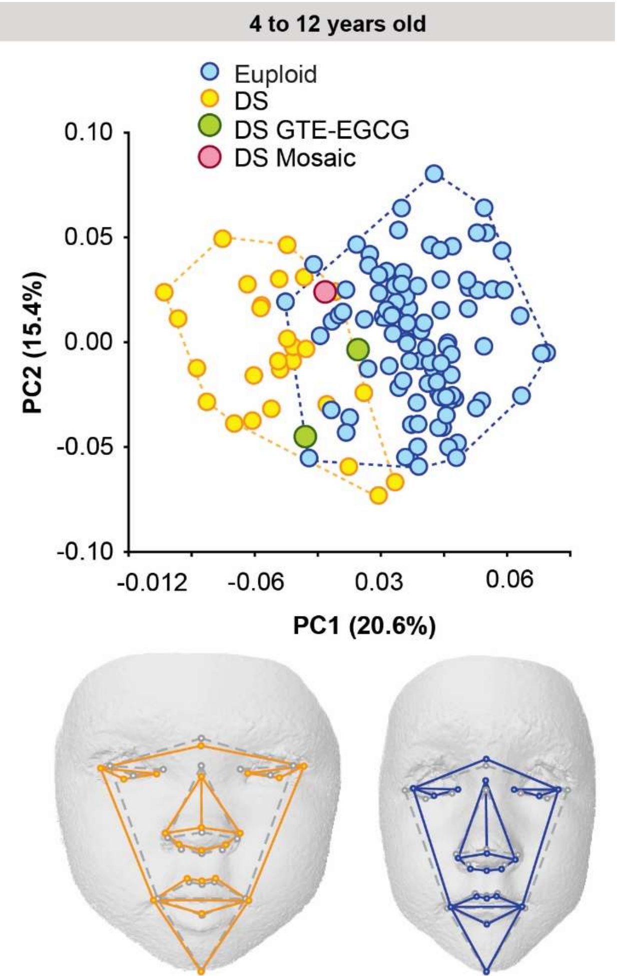

Fig. S8. Principal Component Analyses of the global facial shape variation in children from 4 to 12 years old. Preliminary results showed that mosaic and treated children fell within the overlapping area between the euploid and the Down syndrome range of variation, suggesting a tendency for these children to display milder facial phenotypes. Scatterplot of PC1 and PC2 axes with the corresponding percentage of total morphological variation explained are displayed along each axis. Convex hulls represent the ranges of variation within each group of children. Anterior facial morphings associated with the extreme negative and positive values of PC1 are also shown. Orange solid lines represents facial phenotypes associated with Down syndrome, whereas blue solid line represents facial phenotype associated with euploid condition. Both shapes are compared to average facial shape, represented by a dashed grey line. Facial reconstructions obtained from 3dMD and Agisoft PhotoScan, landmarks recorded with PhotoModeler and morphings performed with Amira.

Table S1. Demographic and clinical details of all children with Down syndrome treated with GTE-EGCG in each age group.

	Sex	Geographical origin	Age	Onset	Duration	Dose	Brand supplement	Composition
13-18 y	M	Europe	17 y	16 y	11 m	532 mg/day	FontUp	Minimum of 94% EGCG
	M	Europe	16 y	15 y	18 m	266 mg/day	FontUp	
	M	Europe	16 y	16 y	2 m	650 mg/day	Megagreen Tea, Life Extension	53.6% EGCG; 4.5% EC, 9.0% ECG, 12.5% EGC
	M	Europe	16 y	16 y	7 m	266 mg/day	FontUp	Minimum of 94% EGCG
**Total**	**4 (0F/4M)**	**4 Europe/ 0 NorthAm**	**16.25 y**	**15.75 y**	**9.5 m**			
4-12 y	M	Europe	6 y	4 y	2 y	266 mg/day	FontUp	Minimum of 94% EGCG
	M	North America	5 y	3 y	2 m	40 mg/day	Green Tea Elite with EGCG by Enzymatic Therapy	57% polyphenols minimum of 39% EGCG
**Total**	**2 (0F/2M)**	**1 Europe/ 1 NorthAm**	**5.5 y**	**3.5 y**	**12.33**			
0-3 y	M	Europe	42 m	30 m	12 m	65 mg/day	FontUp	Minimum of 94% EGCG
	F	North America	26 m	12 m	6 m	26 mg/day	Thorne Green Tea Phytosome	Detailed composition not available
	M	Europe	23 m<br>18 m	14 m	9 m<br>4 m	9mg EGCG/kg/day<br>9mg EGCG/kg/day	FontUp	Minimum of 94% EGCG
	M	Europe	15 m	3 m	12 m	9-13mg EGCG/kg/day	Teavigo Pure Encapsulations	94% EGCG; 0.8% EC, 6.3% ECG, 1.4% EGC
	F	Europe	9 m<br>5 m	1 m	8 m<br>4 m	9mg EGCG/kg/day<br>9mg EGCG/kg/day	FontUp	Minimum of 94% EGCG
**Total**	**5 (+2) (2F/3M)**	**4 Europe/ 1 NorthAm**	**19.7 m**	**12 m**	**7.8 m**			

Note that in the 0-3-year-old group two children were photographed and measured on two separate occasions with 4-5 months in between. All children included in the analyses presented full trisomy 21 and were from Caucasian origin. No mosaic treated cases were included in the analyses.
F: females, M: males, m: month, y: year.
EC: epicatechin, ECG: epicatechin gallate, EGC: epigallocatechin.

Table S2. Definitions of facial landmarks in adult mice (A) and children (B). Landmarks were collected on micro-CT 3D reconstructions of the craniofacial skeleton of adult mice of the Ts65Dn Down syndrome mouse model, and from 3D images of children. See fig. S1 for anatomical reference.

A)	Anatomical location
1	Intersection of the nasal and frontal bones
2	Tip of the nasal bone
3	Anterior-most point at the intersection between premaxilla and nasal bones (right)
4	Anterior-most point at the intersection between premaxilla and nasal bones (left)
5	Center of alveolar ridge over maxillary incisor (right)
6	Center of alveolar ridge over maxillary incisor (left)
7	Intersection of frontal process of maxilla with frontal and lacrimal bones (right)
8	Intersection of frontal process of maxilla with frontal and lacrimal bones (left)
9	Anterior notch on frontal process lateral to infraorbital fissure (right)
10	Anterior notch on frontal process lateral to infraorbital fissure (left)
11	Most inferior point on the premaxilla-maxilla suture (right)
12	Most inferior point on the premaxilla-maxilla suture (left)

B)	Anatomical location
1	Glabella: midpoint between the eyebrows on the median plane
2	Sellion: deepest point of the nasal root
3	Pronasale: most anterior point of the nose tip
4	Subnasale: point where the nasal septum meets the philtrum
5	Labiale superius: midpoint of the vermelion seam of the upper lip
6	Labiale inferius: midpoint of the vermelion seam of the lower lip
7	Gnathion: most inferior point of the chin
8	Endocanthion R: point in the internal lateral commissure of the eye (right)
9	Endocanthion L: point in the internal lateral commissure of the eye (left)
10	Palpebrale inferius R: most inferior medial point of the lower eyelid (right)
11	Exocanthion R: Point in the external lateral commissure of the eye (right)
12	Palpebrale inferius L: most inferior medial point of the lower eyelid (left)
13	Exocanthion L: Point located in the external lateral commissure of the eye (left)
14	Alare R: most lateral point of the nasal wings (right)
15	Subalare R: the facial insertion of the alar base (right)

16	Subalare L: the facial insertion of the alar base (left)
17	Alare L: most lateral point of the nasal wings (left)
18	Chelion R: point located in the labial commissure (right)
19	Crista philtra R: crossing of vermolion line and elevated margin of the philtrum (right)
20	Crista philtra L: crossing of vermolion line and elevated margin of the philtrum (left)
21	Chelion R: point located in the labial commissure (right)
